# Efficient Charge Transport Enables High Efficiency in Dilute Donor Organic Solar Cells

**DOI:** 10.1021/acs.jpclett.1c01219

**Published:** 2021-05-21

**Authors:** Nannan Yao, Jianqiu Wang, Zeng Chen, Qingzhen Bian, Yuxin Xia, Rui Zhang, Jianqi Zhang, Leiqiang Qin, Haiming Zhu, Yuan Zhang, Fengling Zhang

**Affiliations:** † Department of Physics, Chemistry and Biology (IFM), Linköping University, Linköping 58183, Sweden; ‡ School of Chemistry, Beijing Advanced Innovation Center for Biomedical Engineering, Beihang University, Beijing 100191, P. R. China; § State Key Laboratory of Modern Optical Instrumentation, Center for Chemistry of High-Performance & Novel Materials, Department of Chemistry, Zhejiang University, Hangzhou 310027, P. R. China; ∥ Institute for Materials Research (IMO-IMOMEC), Hasselt University, Wetenschapspark 1, 3590 Diepenbeek, Belgium; ⊥ National Center for Nanoscience and Technology, Beijing 100190, P. R. China

## Abstract

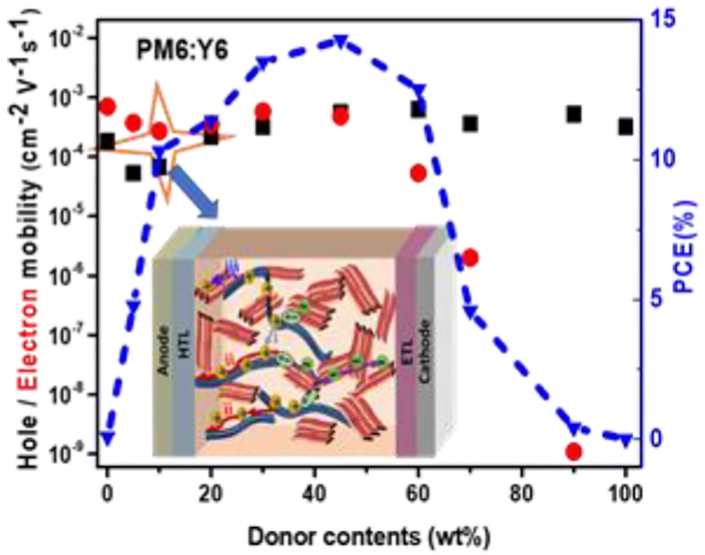

The donor/acceptor weight ratio is crucial for photovoltaic performance of organic solar cells (OSCs). Here, we systematically investigate the photovoltaic behaviors of PM6:Y6 solar cells with different stoichiometries. It is found that the photovoltaic performance is tolerant to PM6 contents ranging from 10 to 60 wt %. Especially an impressive efficiency over 10% has been achieved in dilute donor solar cells with 10 wt % PM6 enabled by efficient charge generation, electron/hole transport, slow charge recombination, and field-insensitive extraction. This raises the question about the origin of efficient hole transport in such dilute donor structure. By investigating hole mobilities of PM6 diluted in Y6 and insulators, we find that effective hole transport pathway is mainly through PM6 phase in PM6:Y6 blends despite with low PM6 content. The results indicate that a low fraction of polymer donors combines with near-infrared nonfullerene acceptors could achieve high photovoltaic performance, which might be a candidate for semitransparent windows.

Organic solar cells (OSCs) based on nonfullerene acceptors (NFAs) have achieved rapid development in recent years due to the tunable energy levels and absorption spectra of NFAs.^1−6^ The power conversion efficiency (PCE) of single-junction OSCs has reached over 18%.^7−9^ In NFA-OSCs, efficient charge separation and low voltage losses can be achieved simultaneously, yielding high short circuit current density (*J*_sc_) and open-circuit voltage (*V*_oc_).^10−12^ The third important parameter fill factor (FF) is strongly affected by the competition between charge transport and recombination. It has been demonstrated that efficient charge transport and suppressed recombination losses are largely related to balanced electron and hole mobility in OSCs.^13,14^ Generally, a continuous interpenetrating network of donor and acceptor is considered to be significant for charge transport.^15^ Therefore, the donor/acceptor (D/A) blend composition is important for charge transport and device performance. Typically, in dilute donor heterojunctions where the average distance between individual donor domain enlarges, that may reduce hopping rate of hole. The unbalanced charge transport can induce a space-charge effect, causing more recombination loss and poor performance.^16^ Interestingly, some previous studies have reported that fullerene based OSCs with very low donor contents could maintain efficient hole transport.^17,18^ It was proposed that hole tunneling could occur between isolated donors,^19,20^ another possible channel is that fullerene acceptors such as PCBM can act as an ambipolar conductor for both electrons and holes in dilute donor solar cells.^17,21,22^

Compared to the clarification on the exclusive role of fullerene acceptors in dilute donor solar cells, charge generation, transport, and recombination in dilute donor devices with NFAs are rarely investigated. Recently, NFA Y6 with the A–D–A′–D–A configuration has received great attention. In the optimized solar cells based on PM6:Y6 (1:1.2, w/w), a high *J*_sc_ exceeding 25 mA cm^–2^ with a low voltage loss (∼0.48 eV) can be achieved simultaneously.^6,23^ A report with single crystal analysis and molecular simulation has highlighted the unique packing in Y6 films can lead to strong electronic coupling between adjacent Y6 molecules and efficient 3-dimensional ambipolar transport networks.^24^ These interesting phenomena raise an important question about hole transport mechanisms in bulk heterojunctions (BHJs) with this emerging family of NFAs, which could contribute to high device performance. Dilute donor solar cells are interesting models to understand the role of acceptors on charge generation, transport and extraction. Therefore, it will be informative to investigate the correlation between photovoltaic performance and donor/acceptor blend composition in NFA-OSCs.

In this work, we studied the photovoltaic behaviors of PM6:Y6 solar cells with various PM6 contents. The OSCs with 10 wt % PM6 yield a high PCE of 10.3% with *J*_sc_ of 18.5 mA cm^–2^ and FF of 0.66. The results of femtosecond transient absorption (TA) spectroscopy indicate efficient charge transfer in dilute donor blend films. Moreover, slow charge recombination and almost field-independent charge extraction in the dilute donor devices were observed in dilute donor solar cells. Importantly, it is found that PM6:Y6 blends even with 10 wt % PM6 exhibit efficient hole mobility and balanced charge transport. By comparing the hole mobility of PM6 diluted at 10 wt % in Y6 and insulating polymers, we conclude that 10 wt % PM6 in PM6:Y6 blends can still retain efficient hole transport pathway through the active layer.

To study the photovoltaic performance, PM6:Y6 solar cells with various PM6 contents were fabricated with a standard structure of ITO/PEDOT:PSS/active layer/PDINO/Al. The photovoltaic parameters extracted from *J–V* curves (Figure S1) are summarized in Figure 1 and Table S1. As shown in Figure 1, the performance of the OSCs exhibit a high tolerance to donor content ranging from 10 to 60 wt %. OSCs with 10 wt% PM6 yield an efficiency over 10% with a high a *J*_sc_ of 18.5 mA cm^–2^ and FF of 0.66. It is worth noting that dilute donor devices show much better performance than the devices with high donor contents, especially *J*_sc_ and FF. To understand the origin of high performance of the OSCs with such low donor contents, charge generation, transport, and recombination were investigated.

**Figure 1:**
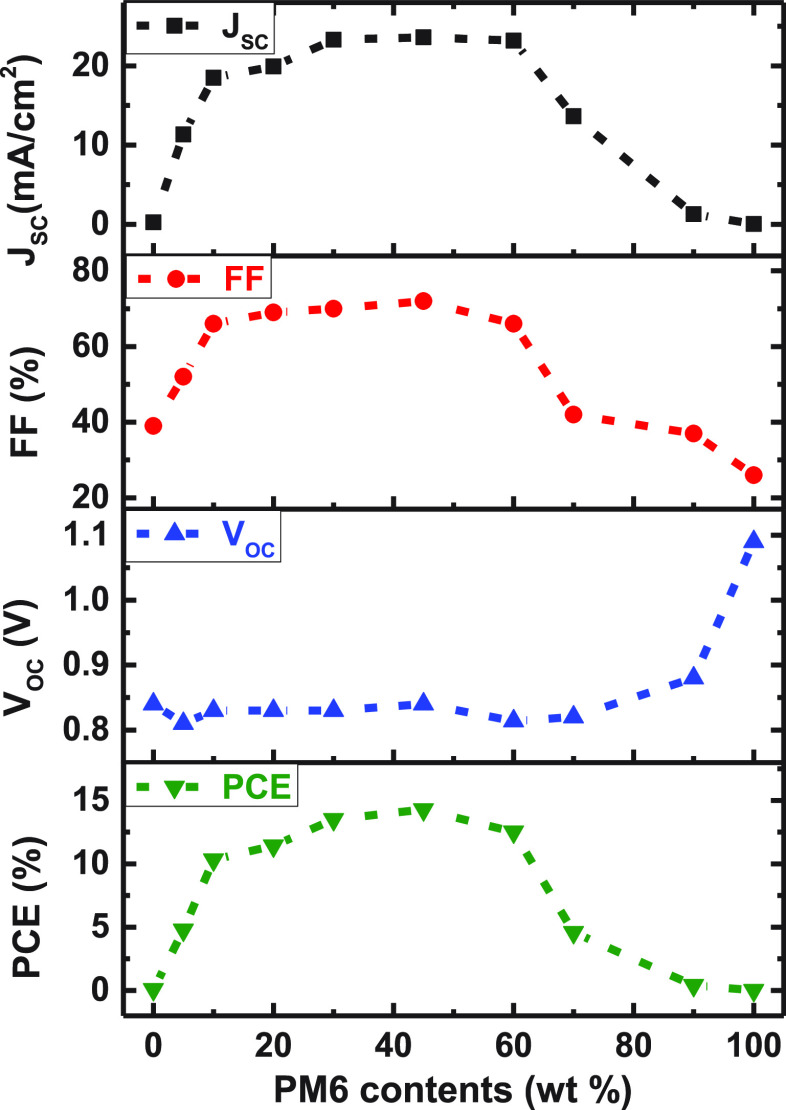
Correlation between photovoltaic performance of PM6:Y6 solar cells and PM6 contents (under 100 mW cm^–2^ solar illumination). The dashed lines are guides to the eyes.

Charge transfer between PM6 and Y6 was investigated by measuring steady-state photoluminescence (PL) spectroscopy (Figure S2). Compared to the PL of pure Y6 films, the large degree of PL quenching in 10 wt % PM6 BHJ indicates efficient interfacial charge transfer. Hole transfer dynamics from Y6 to PM6 was studied with femtosecond TA spectroscopy. In Figure S3 and Figure S4, a 750 nm pump laser was used to selectively excited Y6. The rising kinetics of PM6 ground state bleach in the blend at ∼605 nm reflects the hole transfer process from Y6 to PM6, since photoexcited Y6 shows no TA signal there (Figure S3). The hole transfer process exhibits a biphasic behavior with an ultrafast interfacial hole transfer process and a relatively slower diffusion mediated process.^25−27^ We fit the hole transfer kinetics (Table S2) by a biexponential function
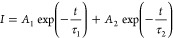
with two lifetimes of τ_1_ and τ_2_ and prefactors of *A*_1_ and *A*_2_.^28^ The initial ultrafast charge transfer process (Figure 2a) indicated by τ_1_ (<250 fs) can be attributed to quantum coherence, which plays a role in the formation of charge carriers^29^ and was experimentally observed in OSCs.^30^ The exciton diffusion process characterized by *A*_2_ and τ_2_ becomes more dominant with the decreasing of PM6 contents. Typically, large amounts of small molecules tend to aggregate,^25^ there would be expected larger domains in 10 wt % PM6 BHJ films. The larger root-mean-square (RMS) of 10 wt % PM6:Y6 films (Figure S5) also indicates strong molecular aggregation, which could lead to oversized domains.^31^ Our previous work has shown that excitons will be created away from the interface and thus spend longer time to diffuse to the interfaces because of the larger domain in BHJ films with low donor contents.^25^ Similarly, the exciton diffusion mediated hole transfer lifetime τ_2_ in PM6:Y6 blends decreases with the amount of donor, as shown in Figure 2b. Here, we also calculated the hole transfer efficiency (HTE) of PM6:Y6 BHJs, as shown in Table S3. A THE of 83% in 10 wt % PM6:Y6 blend reveals efficient hole transfer. In addition, the decay signals in Figure 2a indicate that PM6:Y6 blend films with low donor contents behave slower decay (half-lifetime of 1.45 ns for 5% PM6-based BHJs and 1.5 ns for 10 wt % PM6-based BHJs) than 90 wt % PM6-based BHJs (∼0.35 ns), suggesting slower charge recombination in dilute donor blends. In OSCs, if the charge transport is not efficient, charge recombination will occur after exciton dissociation.

**Figure 2:**
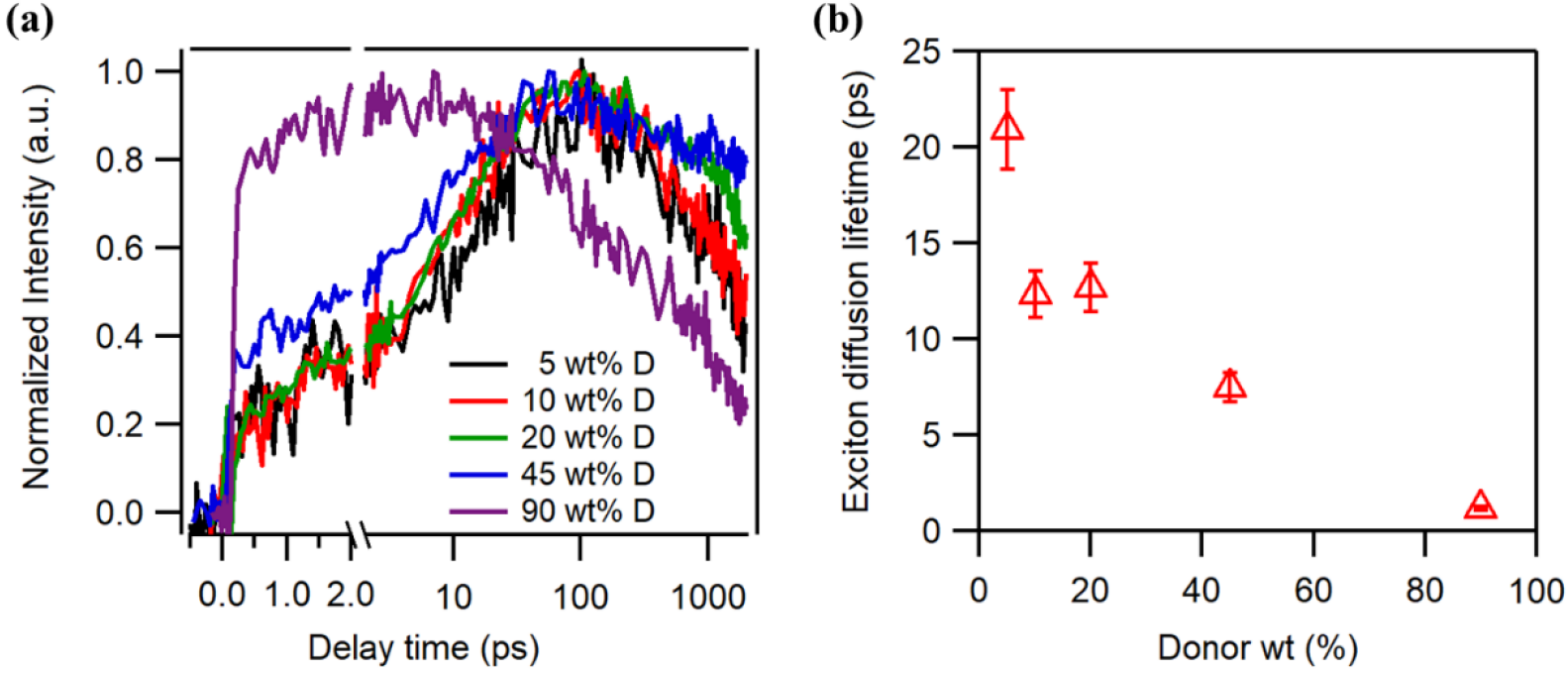
(a) Normalized TA kinetics of hole transfer in PM6:Y6 blend films with various PM6 contents (pump at 750 nm, probe at 605 nm). (b) Exciton diffusion lifetime as a function of PM6 contents (wt %).

To investigate the charge transport, charge carrier mobilities were determined from space charge limited current (SCLC) in single-carrier devices, as shown in Figure S6 and Figure 3. Interestingly, the hole mobilities have little correlation with the PM6 contents, the devices even with 10 wt % PM6 can still exhibit a high electron mobility of 2.7 × 10^–4^ cm^2^ V^–1^ s^–1^ and a hole mobility of 6.8 × 10^–5^ cm^2^ V^–1^ s^–1^ (Table S4), indicating efficient charge transport in such dilute donor structure. However, the electron mobilities show an obvious decrease when PM6 contents is over 60 wt %, the unbalanced charge transport results in a decreased device performance (Figure 1). It is worth noting that pristine Y6 devices show both high electron mobility (6.5 × 10^–4^ cm^2^ V^–1^ s^–1^) and hole mobility (1.8 × 10^–4^ cm^2^ V^–1^ s^–1^), indicating Y6 is an ambipolar conductor for electron and hole.

**Figure 3:**
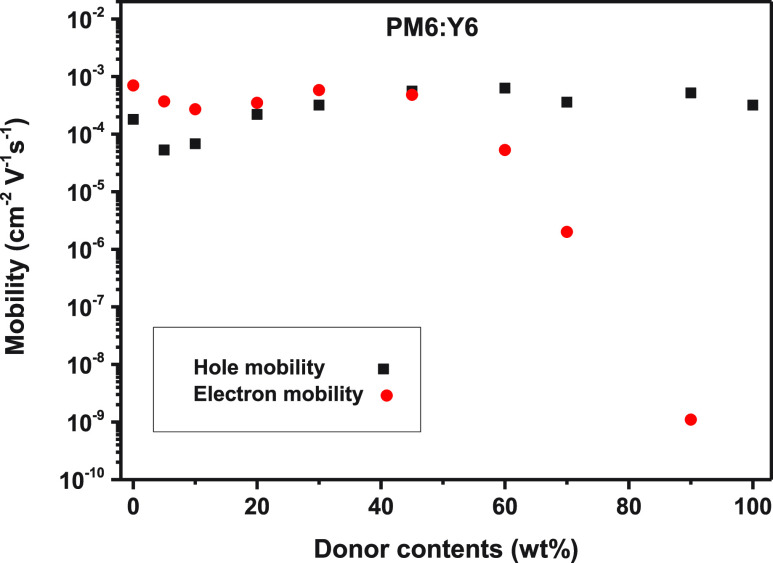
Electron and hole mobilities in PM6:Y6 OSCs with various donor contents extracted from SCLC in single-carrier devices.

To study the charge recombination in device operational conditions, we examined *J*_sc_ under different light intensities. As shown in Figure 4a, the calculated slopes (α) for 10 and 45 wt % PM6 based solar cells are 0.94 and 0.95, indicating smaller bimolecular recombination loss. The relatively low α = 0.76 in the device based on 90 wt % PM6 implies stronger recombination, which indicates that there is the emergence of strong space charges effect in 90 wt % donor devices mainly caused by extremely unbalanced charge transport.^16,32^Figure 4b shows *V*_oc_ dependence on light intensity, the slopes (*n*) of PM6:Y6 devices with 10 and 45 wt % donor are around 1.3, indicating that the recombination is primarily via the bimolecular pathways with a minor role of trap-assisted recombination.^33,34^ The slope of the dilute acceptor device (90 wt % PM6) is very close to unity, which suggests that photogenerated carriers are purely lost via bimolecular recombination. Moreover, transient photovoltage (TPV) measurements were performed on PM6:Y6 solar cells, as shown in Figure 4c. The solar cells with 10 and 45 wt % PM6 exhibit slower recombination with longer decay times of 49.1 and 75.5 μs, respectively; however, the device with 90 wt % PM6 shows much faster charge recombination with a shorter decay time of 31.6 μs, which agrees with the above results.

**Figure 4:**
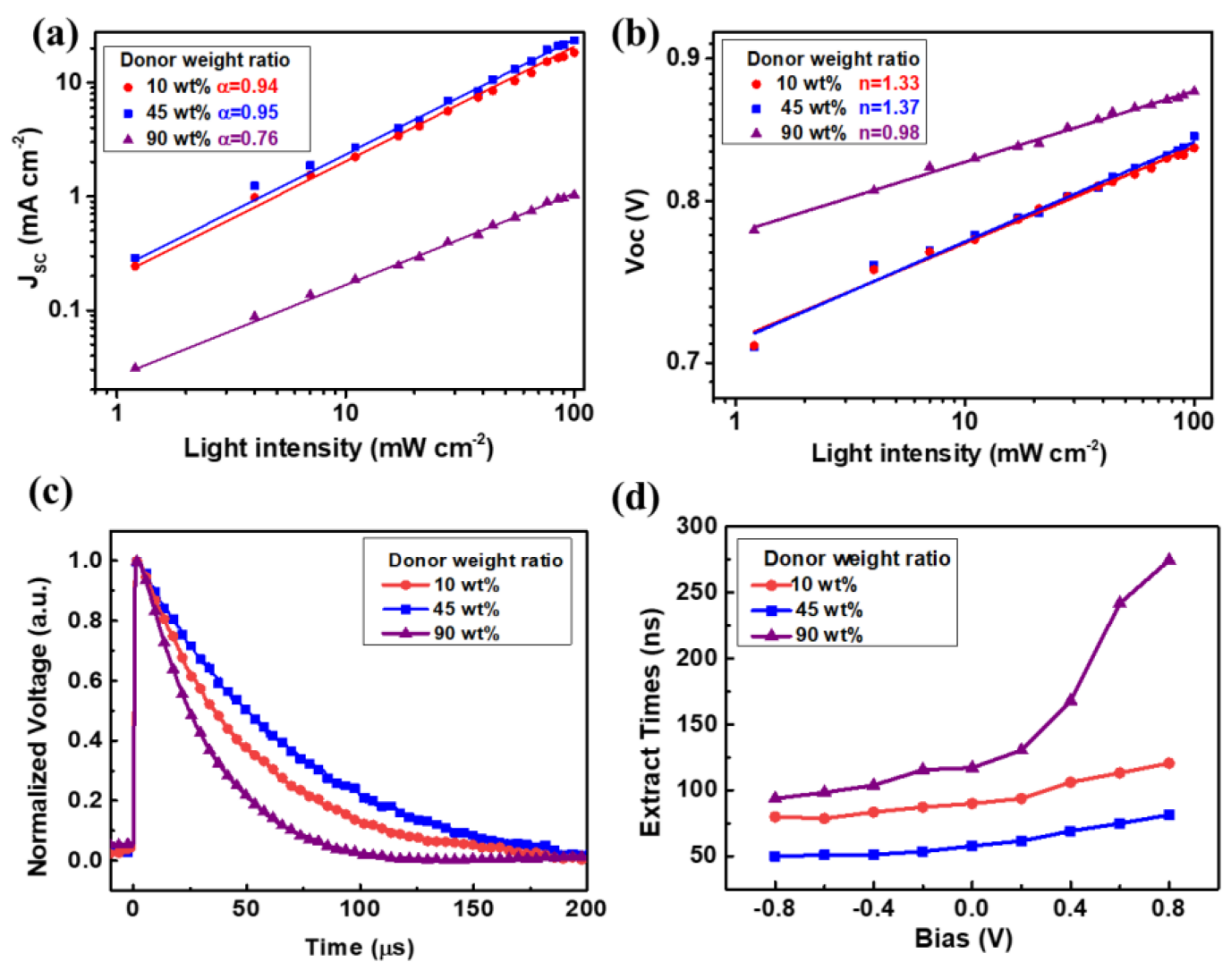
Light intensity-dependent (a) *J*_sc_ and (b) *V*_oc_ characteristics of PM6:Y6 solar cells with various PM6 weight ratios. (c) TPV under open-circuit conditions. (d) Charge extraction time (τ_ext_) as a function of external bias in the studied solar cells determined from TPC measurements.

In the case of extremely unbalanced D/A ratios, a discontinuous interpenetrating network of donor and acceptor might be an important issue for charge transport. Also, a reduced contact area of anode/donor or cathode/acceptor could lead to a low charge extraction efficiency. Here we investigated charge extraction processes by using bias-dependent transient photocurrent (TPC) measurements (Figure S7); charge extraction time versus bias were obtained from TPC with a monoexponential decay model. In Figure 4d, the most notable difference is that, compared to the device with 90 wt % PM6, the solar cells with 10 and 45 wt % PM6 contents show faster and nearly field-independent charge transport and extraction, especially near the low internal field region.

Our previous work demonstrated that charge extraction correlates with bias dependence of the external quantum efficiency (EQE) of OSCs.^14^ Bias-dependent EQE spectra of 10, 45, and 90 wt % PM6-based devices were recorded and are shown in Figure S8. The EQE spectra of devices with 10 and 45 wt % donor contents are almost insensitive to bias, which is consistent with the bias-TPC results. This feature can arise from the suppressed recombination loss at low internal fields, so that photogenerated carriers can be collected more efficiently, yielding high *J*_sc_ and FF in the OSCs with 10 wt % PM6.

Above results demonstrate that the OSCs with 10 wt % PM6 show high and balanced charge transport, which enables slow charge recombination and fast charge extraction. As for the origin of efficient hole transport in dilute donor devices, we assume following hole transport pathways: (i) There is possibility for holes tunneling between isolated donors.^19^ (ii) Holes can transport directly in donor phase. (iii) Y6 may facilitate the hole transport because it is an ambipolar conductor.

To clarity the hole transport mechanism in PM6:Y6 solar cells with 10 wt % PM6, we compared the hole mobilities of PM6:Y6 with PM6:PS (polystyrene) with the same PM6 content because PS is an insulator with very low hole mobility in the order of 10^–8^ cm^2^ V^–1^ s^–1^. As for the results shown in Figure S9 and Table 1, the hole mobilities of PM6:Y6 and PM6:PS with 1:9 weight ratios are in the same order of magnitude, which are comparable to the hole mobility of pristine PM6, indicating the hole transport via the PM6 phase can be a dominant mechanism in 10 wt % PM6 BHJs. To confirm this hypothesis, we measure the hole mobility of PM6 diluted by another insulator poly(methyl methacrylate) (PMMA). In Table 1, PM6:Y6 and PM6:PMMA blends with 10 wt % PM6 also show comparable hole mobilities. Therefore, we conclude that PM6:Y6 blends even with 10 wt % PM6 can still maintain efficient hole transport. Furthermore, according to grazing-incidence wide-angle X-ray scattering (GIWAXS) data (Figure S10), the (100) diffraction peak for PM6 at 0.32 Å^–1^ in in-plane was also observed in 10 wt % PM6:Y6 films, which was most probably induced by the face-oriented Y6 molecules.^35^ This phenomenon reveals that, although in the dilute donor blend, the donor exhibits ordered structure, which can account for high hole mobility in dilute donor devices.

**Table 1:** Hole Mobilities of Pristine PM6, PS, and PMMA and PM6:Y6, PM6:PS, and PM6:PMMA BHJs with 10 wt % PM6

	PM6	Y6	PS	PMMA	PM6:Y6	PM6:PS	PM6:PMMA
μ_h_ [cm^2^ V^–1^ s^–1^]	3.2 × 10^–4^	1.8 × 10^–4^	9.2 × 10^–8^	1.6 × 10^–8^	6.8 × 10^–5^	1.2 × 10^–4^	3.7 × 10^–5^

It is obvious that the dark *J–V* curves of hole-only devices based on pristine Y6 exhibit a trap-filling process, but this phenomenon does not exist in PM6:Y6, PM6:PS, and PM6:PMMA blends (Figure S6 and Figure S9). In addition, hole transfer from donor to acceptor is generally unfavored, because the highest occupied molecular orbital (HOMO) level of Y6 (−5.7 eV) is deeper than that of PM6 (−5.48 eV), which was confirmed by cyclic voltammetry (CV) measurements (Figure S11 and Table S5). Therefore, it is plausible that NFA Y6 has little contribution to hole transport in the dilute donor structure with 10 wt % PM6 (iii), even though Y6 has ambipolar transport characteristics. Given the discussions above, in the dilute donor devices, 10% of PM6 can form efficient hole transport pathways, enabling well-balanced charge transport with sufficiently high mobilities.

To conclude, the performance of PM6:Y6 solar cells with varying stoichiometries was comparatively studied. A high PCE exceeding 10% was achieved in the dilute donor devices (10 wt % PM6) synergized by efficient hole transfer, charge transport, slow charge recombination, and field-insensitive extraction proved with results of TA, TPV, TPC, and bias-dependent EQE. Surprisingly, balanced electron and hole mobilities are achieved in the 10 wt % PM6 OSCs evaluated with SCLC in single carrier devices. By comparing the hole mobility of 10 wt % PM6 mixed with those of Y6, PS, or PMMA, we propose that the dominant hole transport in dilute PM6:Y6 blends is via the PM6 phase. Good performance of dilute donor heterojunctions may open new applications such as for near-infrared photodetectors or semitransparent photovoltaic panels.

## Supporting Information Available

Materials, device fabrication, characterizations, calculation of HTE, *J*–*V* curves, PL, TA, and bias-EQE spectra, kinetics plots, AFM images, 2D GIWAXS patterns, CV, and tables of photovoltaic parameters, hole transfer kinetics, HTE, electron and hole mobilities, and redox potentials and energy levels (PDF)
