# Supplementary material for: Efficient Charge Transport Enables High Efficiency in Dilute Donor Organic Solar Cells
Source: J Phys Chem Lett. 2021 May 21;12(20):5039–44. doi: 10.1021/acs.jpclett.1c01219 (PMC8280696; doi:10.1021/acs.jpclett.1c01219)
Supplement: Supplementary file 1 — jz1c01219_si_001.pdf [file jz1c01219_si_001.pdf]

Supporting Information

Efficient Charge Transport Enables High Efficiency in Dilute Donor Organic Solar Cells

Nannan Yao, [1] *Jianqiu Wang,* [2] *Zeng Chen,* [3] *Qingzhen Bian,* [1] *Yuxin Xia,* [4] *Rui Zhang,* [1] *Jianqi Zhang,* [5] *Leiqiang Qin,* [1] *Haiming Zhu,* [3]* *Yuan Zhang,* [2]* *Fengling Zhang*[1]*

AUTHOR ADDRESS

[1] Department of Physics, Chemistry and Biology (IFM), Linköping University, Linköping, 58183, Sweden

[2] School of Chemistry, Beijing Advanced Innovation Center for Biomedical Engineering, Beihang University, Beijing 100191, P. R. China

[3] State Key Laboratory of Modern Optical Instrumentation, Center for Chemistry of High-Performance & Novel Materials, Department of Chemistry, Zhejiang University, Hangzhou 310027, P. R. China

[4] Institute for Materials Research (IMO-IMOMEC), Hasselt University, Wetenschapspark 1, 3590 Diepenbeek, Belgium

[5] National Center for Nanoscience and Technology, Beijing, 100190, P. R. China

Corresponding Authors

*Haiming Zhu : E-mail: hmzhu@zju.edu.cn

*Yuan Zhang : E-mail: yuanzhang@buaa.edu.cn

*Fengling Zhang : E-mail: fengling.zhang@liu.se

Experimental Section

Materials

PM6, PDINO were purchased from Solarmer, Inc (Beijing). Y6 was purchased from The Hong Kong University of Science and Technology (HKUST). All materials were used as received without additional purification. PEDOT:PSS was purchased from Heraeus (CLEVIOSTM PVP Al 4083). The molecular weight of PM6 used in this work is Mn 45kDa, Mw 97kDa, PDI=2.16.

Device fabrication

Organic solar cells (OSCs) were prepared with conventional structure of ITO/PEDOT:PSS/PM6:Y6/PDINO/Al. The ITO-coated glasses were cleaned by detergent and then treated by TL-1 with a mixture of water, ammonia, and hydrogen peroxide (volume ratio 5:1:1). PEDOT:PSS was spin-coated on ITO at 4000 rpm for 30 s, followed by annealing at 150 °C for 15 min. PM6:Y6 with different donor concentrations were dissolved in chloroform with a total concentration of 16-18 mg/mL. 0.5% CN (volume ratio) was used as additive in blend solution. The active layers were spin-coated on ITO/PEDOT:PSS and then were thermally annealed at 110 °C for 10 min in glovebox. After that PDINO (1.5 mg/mL in methanol) was spin-coated on the active layers at 3000 rpm to get the thickness of 10 nm. Finally, 100 nm of Al was thermally evaporated through shadow mask in a vacuum 1×10^{-6} mbar. The effective device area of devices is 0.047 cm^2. For electron-only devices, ZnO acted as electron transport layer was spin-coated on the ITO substrates and then were thermally annealed at 120 °C for 30 min. The active layers were spin-coated on ITO/ZnO, followed by annealing at 110 °C for 10 min. After spin coating PDINO, Al electrode was deposited to form the configuration of ITO/ZnO/Active layers/PDINO/Al. For hole-only devices (ITO/PEDOT:PSS/Active layer/MoO_3/Ag), MoO_3 and Ag were deposited by evaporation under a pressure ca. 1×10^{-6} mbar.

Characterizations

The Current density-voltage (*J-V*) curves were characterized by a Keithley 2400 source meter with AM 1.5G solar simulator at an intensity of 100 mW/cm^2. The external quantum efficiency (EQE) spectra were recorded using a Newport Merlin lock-in amplifier. Single carrier devices were examined with a Keithley 2400 Source Meter in dark. To determine the mobility, we adopted the widely used space-charge limited current (SCLC) method with the relation.

$$J = \frac{9}{8}\varepsilon_0\varepsilon_r\mu\frac{(V - V_{bi})^2}{d^3}\exp\left(\frac{0.89}{kT}\gamma\left(\frac{V - V_{bi}}{d}\right)^{\frac{1}{2}}\right)$$

ε_0, ε_r are the vacuum permittivity and dielectric constant of semiconductor, respectively, and d is the thickness of active layer. Here, the dielectric constant of semiconductor ε_r is 3.60, the thickness was shown in Table S4, the slope of 2 in double-logarithmic scaled *J-V* characteristics was chosen as the fitting range. For electron-only solar cells based on pristine donors, there is not space-charge region, therefore, we can not get the μ_e of pristine donors.

PL spectra (excitation at 532 nm) were measured by using an Andor Solis SR393i-B spectrograph with a Newton EM-CCD Si array detector.

For femtosecond transient absorption (TA) spectroscopy, the fundamental output from Yb:KGW laser (1030 nm, 220 fs Gaussian fit, 100 kHz, Light Conversion Ltd) was separated to two light beam. One was introduced to NOPA (ORPHEUS-N, Light Conversion Ltd) to produce a certain wavelength for pump beam (here we use 750 nm, below 10 μJ/cm^2/pulse, 30 fs pulse duration), the other was focused onto a YAG plate to generate white light continuum as probe beam. The pump and probe overlapped on the sample at a small angle les s than 10°. The transmitted probe light from sample was collected by a linear CCD array.

Calculation of hole transfer efficiency (HTE) of PM6:Y6 BHJs

HTE (η_{HT}) is determined by ultrafast HT process and diffusion mediated HT process as the following equation:

$$\eta_{HT} = A_1\,\eta_1 + A_2\,\eta_2$$

where A_1 and A_2 represented the percentage of ultrafast HT process and diffusion mediated HT process, respectively. $\eta_1 \approx 100\%$ is the transfer efficiency of ultrafast HT efficiency. The efficiency of diffusion mediated HT process (η_2) is determined by the competition between first-order rate constants of the Y6 exciton recombination to the ground state (k_R) and the hole transfer from the Y6 to PM6 (k_{HT}), as the following equation:

$$\eta_2 = \frac{k_{HT}}{k_{HT} + k_R} = \frac{\tau_R}{\tau_{HT} + \tau_R}$$

Transient photocurrent (TPC) and transient photovoltage (TPV) measurements were performed on a customized transient measurement systems (Physike Technology Co., Ltd) by using a pulsed semiconductor laser S3 (Coherent, Inc.).

Grazing-incidence wide-angle X-ray scattering (GIWAXS) measurements were conducted on a Xenocs-SAXS/WAXS system with an X-ray wavelength of 1.5418 Å. Pilatus 300 K was used as a 2D detector.

Cyclic voltammetry (CV) was measured to study the HOMO and LUMO levels of the films. The thin film spin-coated on ITO substrate was used as working electrode. The counter electrode was platinum wire, and the reference electrode was Ag/Ag^+ electrode. The electrolyte used in this work was acetonitrile contained 0.1 M TBAPF6, scan rate is 50 mV s^{-1}.

$$E_{HOMO} = -(E_{ox,onset} + 4.68)$$

$$E_{LUMO} = (E_{red,onset} + 4.68)$$

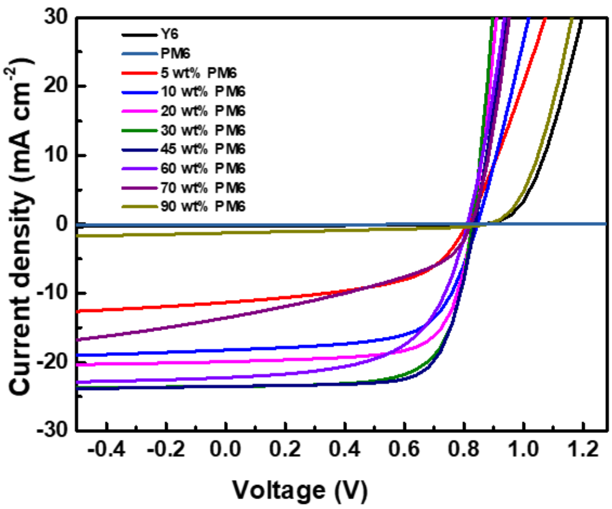

Figure S1. The *J-V* curves of pristine PM6, Y6 solar cells and PM6:Y6 blend-solar cells with various PM6 contents (under AM 1.5 G solar irradiation).

Table S1. Photovoltaic parameters of organic solar cells based on PM6:Y6 with different PM6 contents.

PM6:Y6 PM6 content [wt%]	J_{SC} [mA cm^{-2}]	V_{OC} [V]	FF	PCE[a] [%]
0	0.25	0.84	0.39	0.08 (0.07±0.01)
5	11.35	0.81	0.52	4.78 (4.47±0.31)
10	18.5	0.84	0.66	10.3 (10.22±0.09)
20	19.93	0.83	0.69	11.41 (11.23±0.18)
30	23.30	0.83	0.7	13.54 (13.41±0.13)
45	23.61	0.84	0.72	14.28 (14.12±0.16)
60	23.20	0.82	0.65	12.36 (12.28±0.08)
70	13.65	0.82	0.41	4.59 (4.45±0.14)
90	1.27	0.88	0.37	0.41 (0.36±0.05)
100	0.03	1.09	0.26	0.01 (0.009±0.001)

[a] The data in brackets are average values with standard deviation from 10 devices.

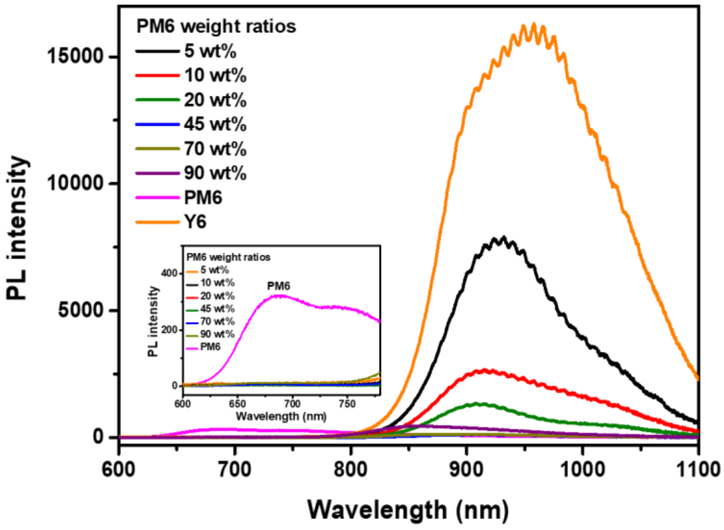

Figure S2. PL spectra of PM6:Y6 BHJ films with different PM6 contents under 532 nm excitation. The insert shows the PL signals of PM6. The PL spectra of the BHJs have been normalized by the absorbance of Y6 in the BHJs.

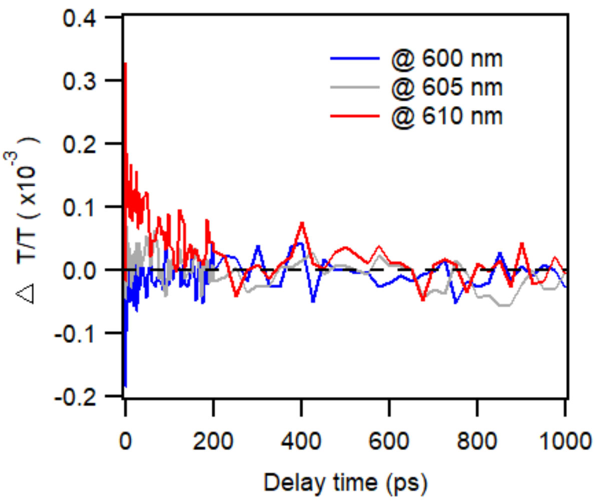

Figure S3. Kinetics of 600 nm, 605 nm and 610 nm in neat Y6 film.

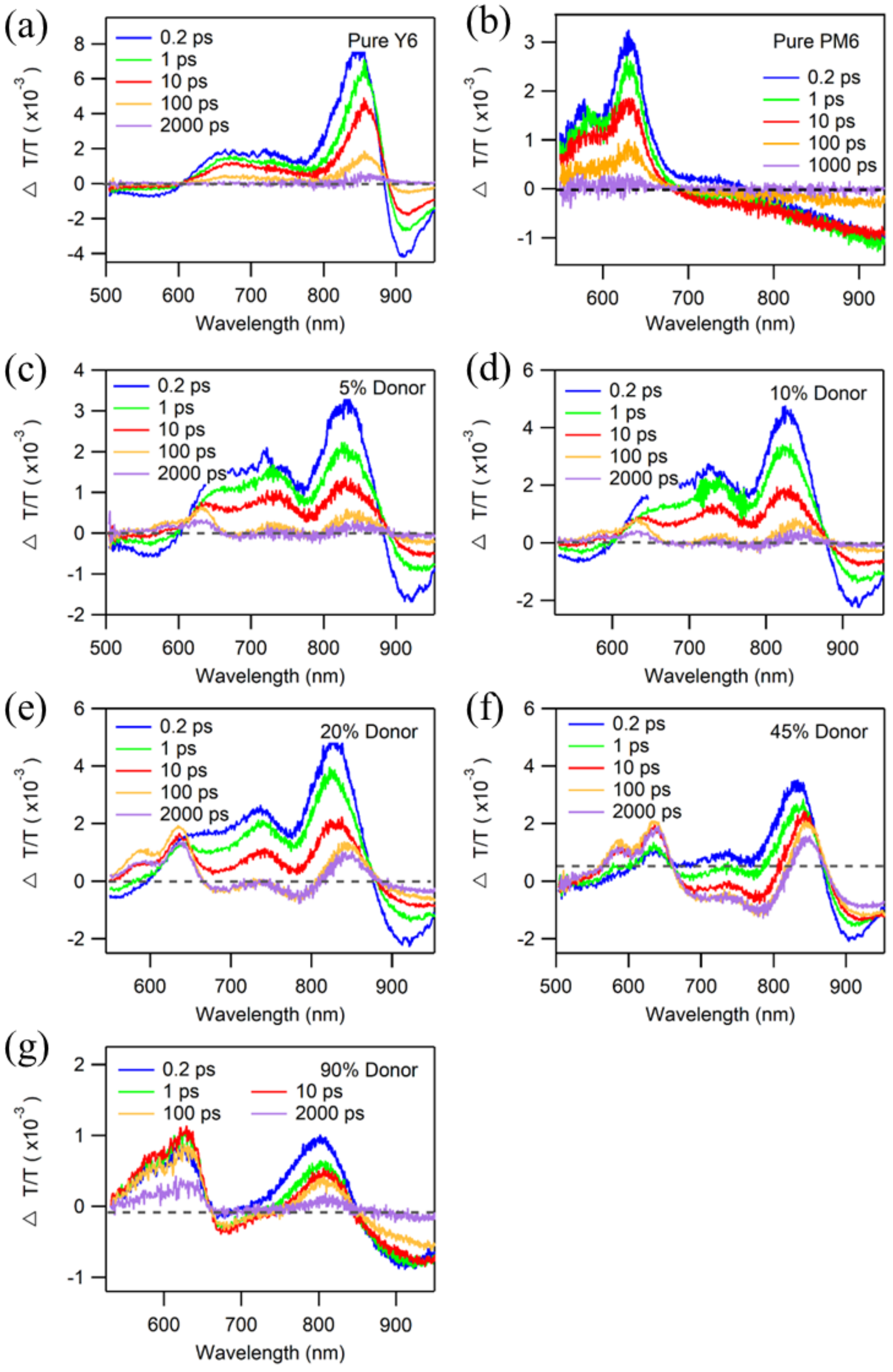

Figure S4. TA spectroscopy (a) Y6, (b) PM6, and PM6:Y6 BHJ films with PM6 contents of (c) 5 wt%, (d) 10 wt%, (e) 20 wt%, (f) 45 wt% and (g) 90 wt% at different delay time.

Table S2. Hole transfer kinetics in PM6:Y6 blends

PM6:Y6<br>PM6 content	A_1	τ_1 [ps]	A_2	τ_2 [ps]	Hole transfer time [ps]
5 wt%	26.7%	0.21±0.02	73.3%	20.91±2.09	15.38±1.54
10 wt%	28.6%	0.24±0.02	71.4%	12.34±1.23	8.88±0.88
20 wt%	32.7%	0.25±0.03	67.3%	12.68±1.27	8.61±0.86
45 wt%	45.2%	0.11±0.01	54.8%	7.48±0.75	4.15±0.42
90 wt%	85.5%	0.09±0.01	14.5%	1.15±0.12	0.24±0.02

Table S3 (a). Diffusion mediated HTE (η_2) of different BHJ films as a function of PM6 contents.

Samples	τ_R	
Neat Y6	42.08±4.21 ps	
PM6 content	τ_{HT}	η_2
5 wt%	20.91±2.09	66.8%
10 wt%	12.34±1.23	77.3%
20 wt%	12.68±1.27	76.8%
45 wt%	7.48±0.75	84.9%
90 wt%	1.15±0.12	99.6%

Table S3 (b). HTE (η_{HT}) of different BHJ films as a function of PM6 ratio.

PM6 content	A_1	A_2	η_2	η_{HT}
5 wt%	26.7%	73.3%	66.8%	75.6%
10 wt%	28.6%	71.4%	77.3%	83.8%
20 wt%	32.7%	67.3%	76.8%	84.4%
45 wt%	45.2%	54.8%	84.9%	91.7%
90 wt%	85.5%	14.5%	99.6%	99.9%

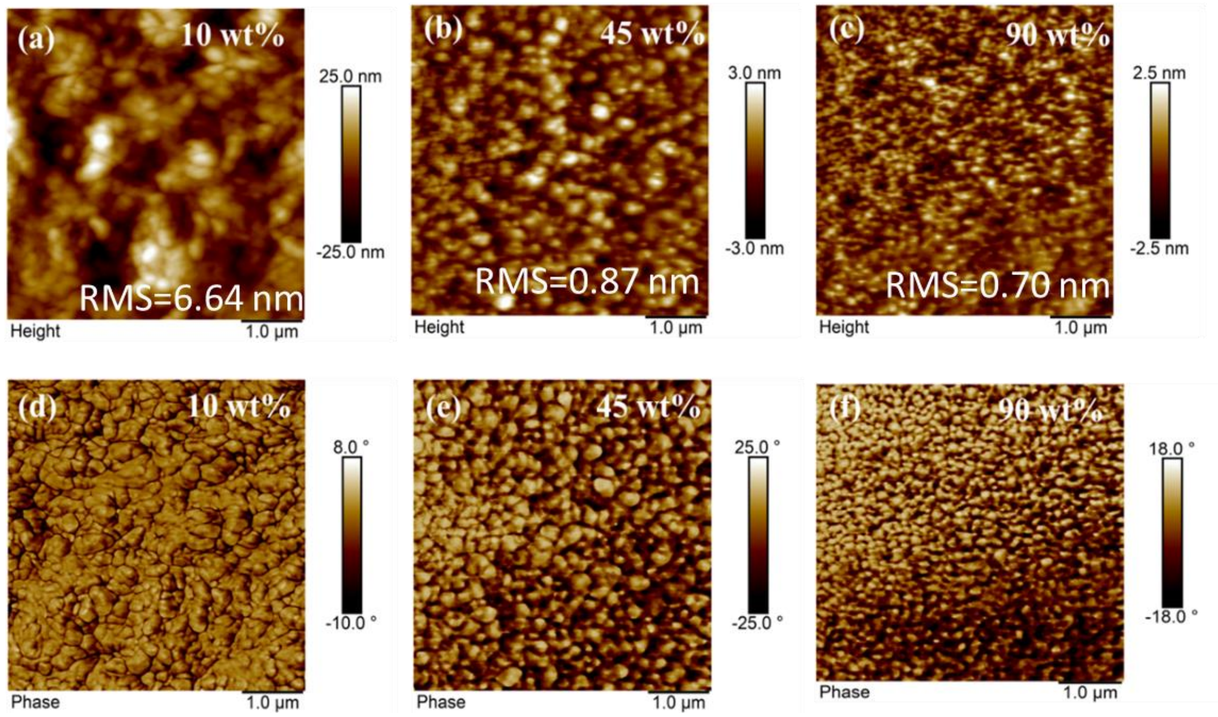

Figure S5. Height (a-c) and phase (d-f) images of PM6:Y6 with 10 wt%, 45 wt% and 90 wt% PM6 by captured by AFM in the tapping-mode (5×5 μm).

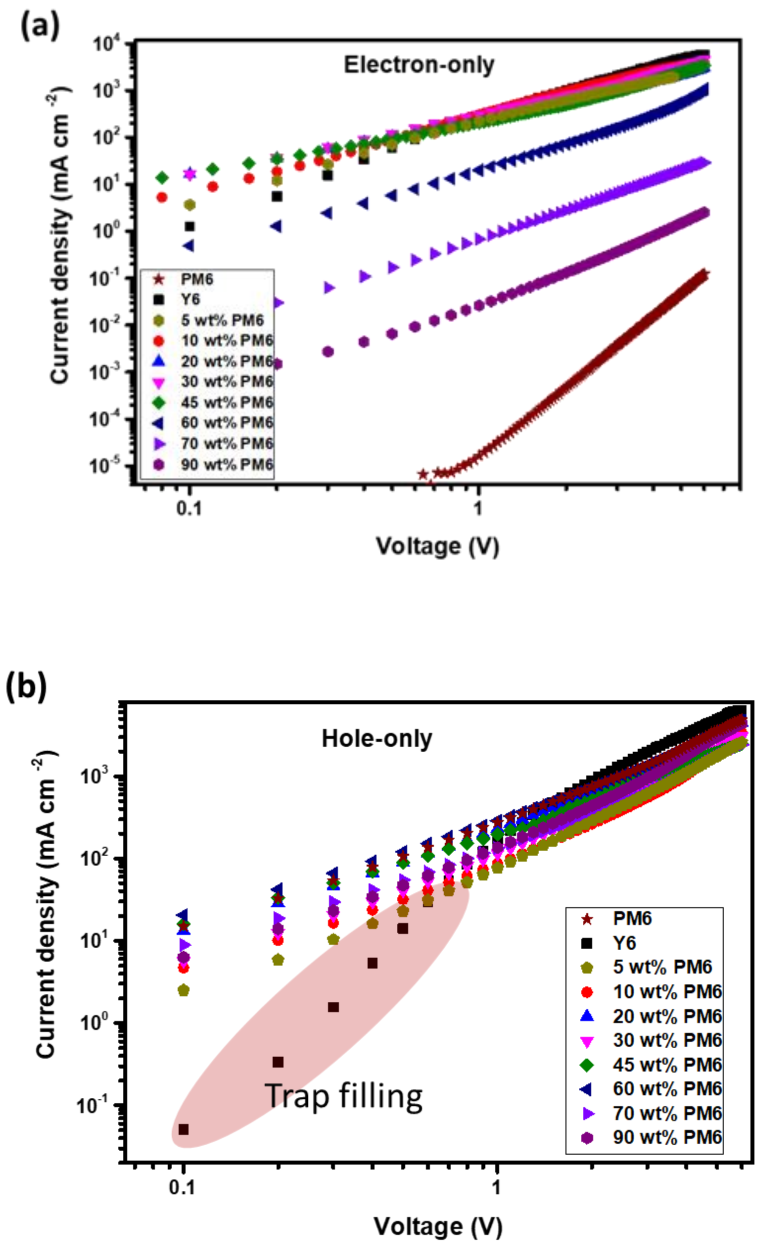

Figure S6. Dark *J-V* curves of (a) electron-only and (b) hole-only devices based on pristine PM6, Y6 and PM6:Y6 BHJs measured at room temperature. There is an obvious trap-filling process in pristine Y6-based hole-only devices, as the red region shown in (b).

Table S4. Electron and hole mobilities of PM6:Y6 solar cells with different PM6 contents.

Materials	Thickness [nm]	μ_h [cm^2 V^{-1} s^{-1}]	μ_e [cm^2 V^{-1} s^{-1}]	μ_e/μ_h
PM6	100	3.2×10^{-4}	/	/
Y6	90	1.8×10^{-4}	6.5×10^{-4}	3.6
5 wt% PM6	100	5.3×10^{-5}	3.7×10^{-4}	6.9
10 wt% PM6	120	6.8×10^{-5}	2.7×10^{-4}	3.9
20 wt% PM6	110	2.2×10^{-4}	3.5×10^{-4}	1.6
30 wt% PM6	120	3.2×10^{-4}	5.8×10^{-4}	1.8
45wt% PM6	120	5.6×10^{-4}	4.8×10^{-4}	0.85
60wt% PM6	120	6.3×10^{-4}	5.3×10^{-5}	0.08
70wt% PM6	130	3.6×10^{-4}	2.0×10^{-6}	5.5×10^{-3}
90 wt% PM6	130	5.2×10^{-4}	1.1×10^{-9}	2.1×10^{-6}

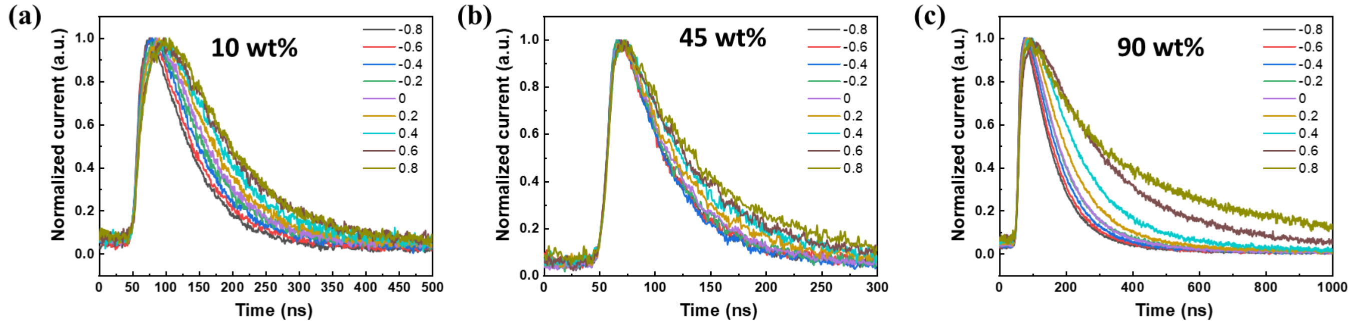

Figure S7. Normalized photocurrent decay kinetics of PM6:Y6 solar cells with different amount of PM6. (a) 10 wt%, (b) 45 wt% and (c) 90 wt%.

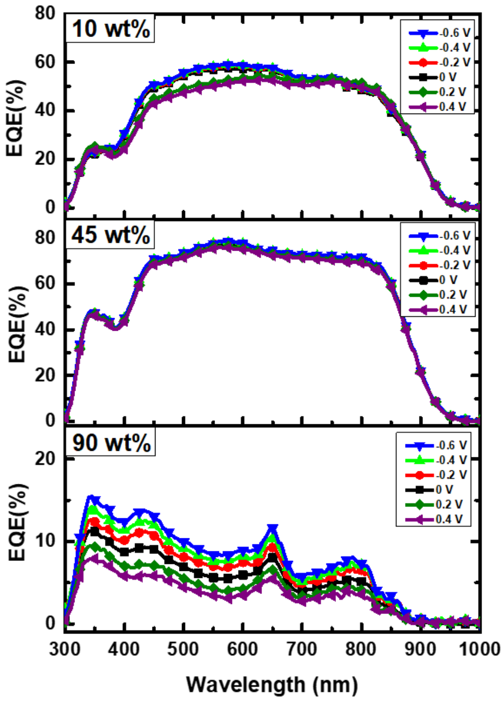

Figure S8. Bias-EQE spectra of PM6:Y6 solar cells with different amount of PM6: 10 wt%, 45 wt% and 90 wt%.

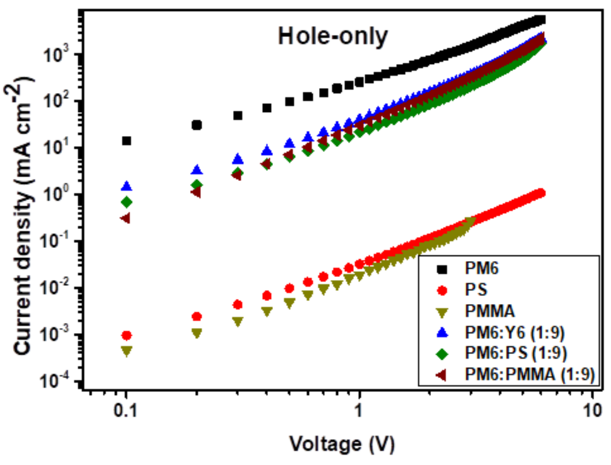

Figure S9. Dark *J-V* curves of hole-only devices based on pristine PM6, PS and PMMA, PM6:Y6, PM6:PS and PM6:PMMA BHJs with 1:9 weight ratios.

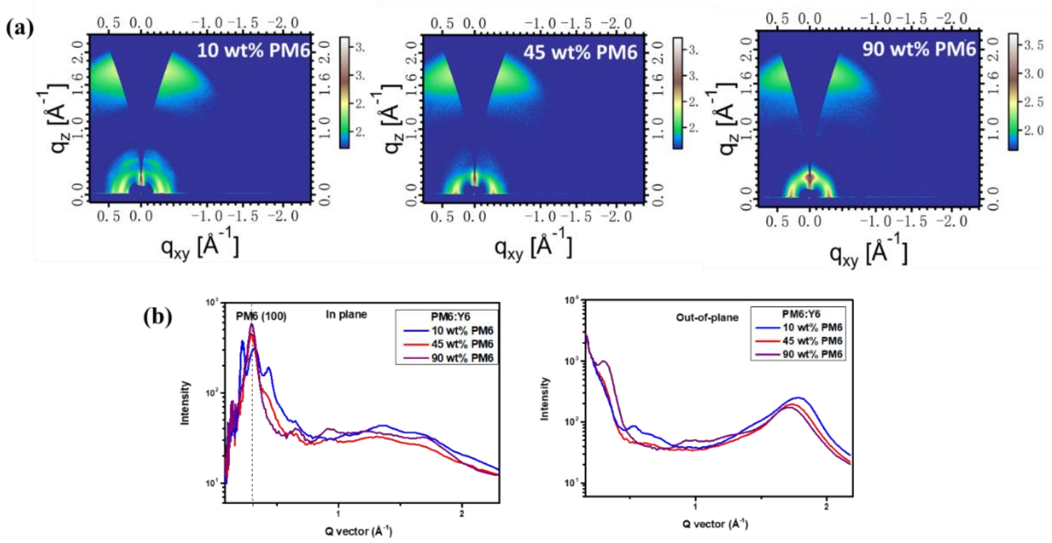

Figure S10. (a) 2D GIWAXS patterns and (b) in-plane and out-of-plane of PM6:Y6 blend films with 10 wt%, 45 wt%, 90 wt% PM6.

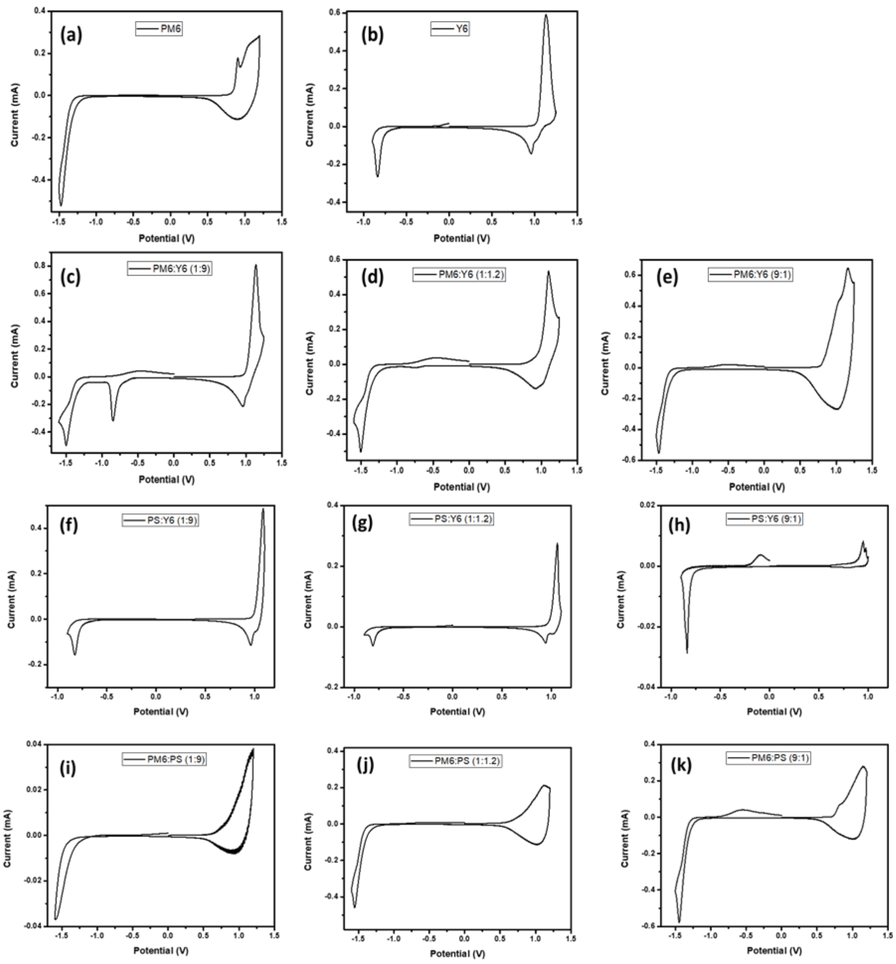

Figure S11. Cyclic voltammetry of (a) neat PM6 and (b) Y6 film; (c-e) PM6:Y6 blend films with D:A ratios of 1:9 (10 wt% PM6), 1:1.2 (45 wt% PM6), 9:1 (90 wt% PM6) ; (f-h) PS:Y6 blend films with D:A ratios of 1:9, 1:1.2, 9:1; (i-k) PM6:PS blend films with D:A ratios of 1:9, 1:1.2, 9:1.

Table S5. Redox potentials and energy levels of PS:Y6, PM6:PS and PM6:Y6 blends.

	Blend ratio	E_{ox}^{onset} vs Ag/Ag^{+} (V)	HOMO (eV)	E_{red}^{onset} vs Ag/Ag^{+} (V)	LUMO (eV)
PM6	/	0.80	-5.48	-1.26	-3.42
Y6	/	1.02	-5.70	-0.75	-3.93
PS:Y6	1:9	1.00	-5.68	-0.75	-3.93
	1:1.2	0.97	-5.65	-0.74	-3.94
	9:1	0.89	-5.57	-0.75	-3.93

PM6:PS	1:9	0.78	-5.46	-1.28	-3.4
	1:1.2	0.75	-5.43	-1.32	-3.36
	9:1	0.74	-5.42	-1.27	-3.41
PM6:Y6	1:9	1.00	-5.68	-1.30	-3.38
	1:1.2	0.94	-5.62	-1.30	-3.38
	9:1	0.82	-5.50	-1.28	-3.40
